# Amoxicillin Resistance: An In Vivo Study on the Effects of an Approved Formulation on Antibiotic Resistance in Broiler Chickens

**DOI:** 10.3390/ani15131944

**Published:** 2025-07-01

**Authors:** Ádám Kerek, Ábel Szabó, Ákos Jerzsele

**Affiliations:** 1 Department of Pharmacology and Toxicology, University of Veterinary Medicine, István utca 2, 1078 Budapest, Hungary; szabo.abel@student.univet.hu (Á.S.); jerzsele.akos@univet.hu (Á.J.); 2 National Laboratory of Infectious Animal Diseases, Antimicrobial Resistance, Veterinary Public Health and Food Chain Safety, University of Veterinary Medicine, 1078 Budapest, Hungary

**Keywords:** amoxicillin, antimicrobial resistance genes (ARGs), broiler chicken, gut microbiota, resistome, metagenomics, low-dose antibiotics, One Health

## Abstract

**Background:** Antimicrobial resistance (AMR) is a growing global concern in poultry production, where antibiotic use can disrupt gut microbiota and enrich antimicrobial resistance genes (ARGs). **Objectives:** This study aimed to assess the in vivo effects of a veterinary-approved amoxicillin formulation on gut microbiome composition and ARG profiles in broiler chickens. **Methods:** A total of 120 Ross-308 broiler chickens were randomly allocated into 12 experimental groups (*n =* 10 per group), with three replicates per treatment. Birds received either full-dose (1×), a subtherapeutic quarter-dose (¼×) of amoxicillin, a placebo (starch), or no treatment. Cloacal swabs were collected on days 0, 14, and 28 for shotgun metagenomic sequencing. One-way ANOVA was used to evaluate treatment effects on body weight, with significant differences observed from day 14 onward (*p* < 0.0001). **Results:** The ¼× dose caused a more pronounced microbiome shift than the 1× dose, with a marked reduction in Pseudomonadota and increase in Bacillota and Bacteroidota. ARG abundance declined in the ¼× group (from 1386 to 1012). While TEM-type ESBL genes were ubiquitous, *CTX-M-1* emerged only after ¼× treatment. Worryingly, 20 types of vancomycin resistance genes were detected across all samples. Plasmid-borne ARGs and mobile genetic elements decreased in the ¼× group. **Conclusions:** Even subtherapeutic antibiotic exposure significantly reshapes the gut microbiota composition and ARG landscape, highlighting the need for refined risk assessments and microbiome-conscious antimicrobial policies in poultry farming.

## Simple Summary

Antimicrobial resistance (AMR) represents one of the most pressing global public health threats. In broiler chickens, the use of antibiotics exerts not only therapeutic effects but also has the potential to significantly alter the composition of the gut microbiota and the abundance of antimicrobial resistance genes (ARGs). The aim of this study was to evaluate the impact of a veterinary formulation containing amoxicillin—approved for use in broiler chickens—on the gut microbiome and resistome profile under different dosing regimens. Our results revealed that a one-quarter therapeutic dose caused substantial shifts in microbial diversity and the relative abundance of specific bacterial taxa, while also significantly reducing the total abundance of antimicrobial resistance genes. In contrast, the full therapeutic dose induced fewer detectable changes. Notably, vancomycin resistance genes were consistently detected in all treatment groups, and their total numbers remained high or even increased in some cases. These findings support the growing body of evidence that the effects of antibiotics extend beyond clinical efficacy; they highlight the importance of considering ecological consequences when administering such agents in livestock production.

## 1. Introduction

The widespread use of antibiotics—whether in human medicine, agriculture, or veterinary practice—contributes to the selection of bacterial populations with enhanced mutational adaptability, increasing the likelihood of resistance development [1,2]. Various environmental stressors such as antibiotics, heavy metals, and ultraviolet radiation can induce oxidative stress which in turn facilitates the horizontal transfer and co-selection of resistance genes [3]. Antibiotic administration has been shown to elevate the resistome of the gut microbiota [4], inducing not only target-specific [5], but also cross-resistance mechanisms [6]. Growth-promoting agents used historically in livestock, including chlortetracycline and bacitracin [7], have played a substantial role in the dissemination of diverse resistance genes [8]. Clinical and experimental data indicate that the use of certain antibiotics—such as enrofloxacin—may lead to resistance against multiple other drug classes, including amoxicillin, sulfonamides, and tetracyclines [9].

Antimicrobial resistance (AMR) poses a major global public health and economic challenge. The spread of multidrug-resistant (MDR) pathogens limits therapeutic options, increases mortality rates, and imposes significant economic burdens both in Europe [10] and the United States [11]. According to the World Health Organization (WHO), the global incidence of several forms of antimicrobial resistance in human clinical settings increased by 15% between 2017 and 2020 [12]. AMR-related deaths already exceed 700,000 annually and are projected to reach 10 million per year by 2050 [13,14]. These alarming trends underscore the importance of addressing AMR not only in human medicine but also in veterinary and agricultural sectors, where antibiotic use may contribute to the selection and spread of resistance.

Broiler chickens represent one of the world’s most important sources of animal protein, with global production experiencing continuous growth [15]. In 2019, global egg production reached approximately 73.2 million metric tons [16] and the global chicken meat output is projected to reach 8.14 billion tons by 2025 [17]. As the global human population increases and diets change, the demand for poultry meat and eggs continues to rise. Over the past 60 years, the amount of poultry meat produced has surged by 1500% [18], with a 2.6% increase reported in 2020 alone [19]. Due to its critical role in food security and the global economy, broiler production is associated with substantial antibiotic use worldwide. This practice is prevalent in both high- and middle-income countries, where maximizing feed conversion efficiency and minimizing mortality remain priorities. For instance, in countries such as Mexico [20], antibiotic application is common due to especially favorable feed conversion ratios and intensive production systems [21]. In light of rising antimicrobial resistance and regulatory pressure to reduce antibiotic usage in food-producing animals, alternative strategies—including probiotics, prebiotics, phytochemicals, and competitive exclusion cultures—are increasingly being explored to maintain animal health and productivity without contributing to resistance development [22,23,24,25,26,27,28,29,30,31].

Multiple studies have demonstrated that *Escherichia coli* and *Salmonella* isolates from poultry can be resistant to a wide range of β-lactam antibiotics [32]. Furthermore, the use of amoxicillin has been linked a co-selection of resistance genes [33,34] to other antimicrobial classes, such as cephalosporins [35], tetracyclines, sulfonamides, and aminoglycosides [36].

Alarmingly, plasmids may carry genes conferring resistance to multiple drug classes. Reports have documented the co-occurrence [37] of *mcr-1* [38] and ESBL genes (e.g., *CTX-M*) in *E. coli* [39] from poultry in Laos, Belgium, and China [40]. Resistance patterns often involve cross-resistance between cephalosporins and antimicrobials such as trimethoprim-sulfamethoxazole, nalidixic acid [41], aminoglycosides, and quinolones [42,43,44].

Beyond *E. coli*, multidrug resistance (MDR) is widespread in other pathogens. *Salmonella Saintpaul* plasmids may encode resistance to nine antibiotic classes [45]; in *Acinetobacter* spp., over 50% are MDR, with ~10% being extensively drug-resistant (XDR) [46]. ESBL-producing *Salmonella Heidelberg* strains have shown resistance to at least eight antimicrobial classes [47], while in China, 17% of *Salmonella* strains were resistant to all tested drugs except carbapenems [48].

The intestinal microbiota of broilers is highly dynamic and sensitive to antibiotic exposure. Colonization begins with facultative anaerobes [49], followed by obligate anaerobes [50], with diversity increasing until about six weeks of age [49,51]. The crop, ileum, and cecum harbor distinct microbial populations influenced by feed content and host physiology [52]. Dominant genera include *Bacteroides*, *Clostridium*, *Oscillospira*, *Ruminococcus*, and *Coprococcus* [53].

Antibiotics significantly alter this ecosystem. For example, penicillin-treated broilers exhibit a higher Firmicutes–Bacteroidetes ratio [51], potentially contributing to growth promotion [54]. Other studies report reduced *Lactobacillus*, *Enterobacteriaceae*, and *Bifidobacterium* levels in the ileum, alongside beneficial reductions in zoonotic *Campylobacter* spp. [53].

Therefore, the objective of this study was to investigate the in vivo impact of a veterinary amoxicillin-containing medicinal product on gut microbiome composition and the development of antimicrobial resistance genes (ARGs) in broiler chickens.

## 2. Materials and Methods

### 2.1. Animal Experimentation Conditions

The animal experiment was conducted at the animal facility of the Department of Pharmacology and Toxicology. According to the statement of the Institutional Animal Welfare Committee of the University of Veterinary Medicine Budapest, and in accordance with Act No. 28 of 1998 §3(9), as well as the Government Decree 40/2013 (II.14) §1(4). The experimental procedures were not classified as interventions that adversely affect animal welfare. Therefore, no project authorization request was required by the competent animal welfare authority. Importantly, the antibiotic formulation used was authorized for administration to broiler chickens, and the applied dosages did not exceed the approved therapeutic levels.

A total of 120 Ross 308 broiler chickens were included in the study. Body weights were measured on the day of hatch and subsequently on days 7, 14, 21, and 28. The animals were randomly allocated into 12 groups, each consisting of 10 birds, and housed in separate cages. Three biological replicates were established for each treatment condition to enable statistical comparison. Birds received either the amoxicillin-containing formulation, a starch-based placebo, or no treatment for 10 consecutive days starting on day 21, in accordance with the manufacturer’s instructions (Table 1).

All birds were housed in cages within a licensed animal facility, fully compliant with prevailing animal welfare and ethical regulations. The experiment followed an all-in/all-out system with strict disinfection and biosecurity protocols in place. Environmental parameters such as temperature, humidity, and lighting were maintained according to the Ross 308 Broiler Management Guidelines. The ambient temperature was initially set at 30 °C and gradually reduced to 20 °C by the end of the trial. A stepped lighting program was applied 24 h of light, then 23 h of light and 1 h of darkness during the next five days, then progressively adjusted to 18 h of light and 6 h of darkness by the end of the grow-out period. Relative humidity was maintained between 60 and 70% (Supplementary Table S2). Birds had unrestricted access to antibiotic-free commercial feed and clean drinking water. Each treatment group was housed separately to avoid any cross-contamination. The feeding regime and the chemical composition of the feed are provided in the Supplementary Table S2.

To analyze the composition and dynamics of the gut microbiota, cloacal fecal sampling was performed upon arrival (one-day old) to assess baseline bacterial populations and antimicrobial resistance gene carriage. Additional fecal samples were collected on the day prior to antibiotic treatment and on the day following the 10-day treatment period.

The antibiotic was administered in accordance with the manufacturer’s recommendations, at a daily dose of 20 mg amoxicillin per kg of body weight (BW), which corresponds to 4 g of Vetrimoxin (Tolnagro Ltd., Szekszárd, Hungary) per 100 kg BW. Birds in groups 4–6 received the full therapeutic dose, while those in groups 1–3 were administered one-quarter of this amount. The calculated daily doses and treatment regimens are summarized in Table 1, which provides a clear overview of the experimental groups, dosing schemes, and sampling time points to facilitate understanding of the study design. The daily dose was dissolved in 30 mL of sterile physiological saline and delivered via crop gavage at a volume of 1 mL per bird.

The placebo used in groups 7–9 consisted of starch—the excipient in Vetrimoxin—at a dose equivalent to 50% of the therapeutic powder mass, calculated accordingly. Treatment was initiated at 21 days of age and continued for 10 days, with the birds reaching 30 days of age at the end of the dosing period. Fecal sample collection was performed 15 days after the final antibiotic administration, when the birds were 45 days old.

According to the manufacturer’s summary of product characteristics, the applied formulation—Vetrimoxin 50 oral powder for veterinary use (A.U.V.)—has a withdrawal period of 3 days for edible tissues in chickens.

### 2.2. Metagenomic Analysis

Fecal samples were sequenced using an Illumina NextSeq 500 (Illumina Inc., San Diego, CA, USA) platform [55]. Genomic DNA was extracted according to the manufacturer’s instructions using the QIAamp DNA Stool Mini Kit (Qiagen, Hilden, Germany). DNA libraries were prepared using the Nextera XT DNA Library Preparation Kit (Illumina, San Diego, CA, USA). For indexing of DNA fragments, we employed the Nextera XT Index Kit v2 Set A (Illumina, San Diego, CA, USA). Final sequencing was performed on an Illumina NovaSeq 6000 (Illumina Inc., San Diego, CA, USA) platform with paired-end 150 bp reads (PE150) [56].

### 2.3. Bioinformatic Analysis

Initial quality control of the raw sequencing reads was performed using FastQC (v0.11.9) [57]. Low-quality and adapter-contaminated reads were filtered with TrimGalore (v0.6.6) [58]. High-quality reads were then assembled into contigs using MEGAHIT (v1.2.9) [59] and contig quality was assessed with QUAST (v5.0.2) [60].

To eliminate host-derived sequences, reads were mapped to the *Gallus gallus* reference genome (NCBI accession: GRCg6a) using Bowtie2 (v2.3.4.1) [61]. Taxonomic classification of reads was performed with Kraken2 (v2.0.8) [62], referencing the NCBI nucleotide database [63]. All downstream microbial community analyses were conducted in R (v3.6.1) [64], utilizing the phyloseq (v1.28.0) [65] and microbiome (v1.8.0 ) [66] packages.

Bacterial-origin contigs were assembled separately using metaSPAdes (v3.13.0) [67]. Open reading frames (ORFs) were predicted with Prodigal (v2.6.3) [68]. Identification of antibiotic resistance genes (ARGs) within ORFs was carried out using the Resistance Gene Identifier (RGI) tool (v5.1.0) against the Comprehensive Antibiotic Resistance Database (CARD). Only genes meeting the STRICT threshold criteria of CARD and demonstrating ≥95% sequence identity and coverage were retained for analysis.

To assess the potential mobility of detected ARGs, we employed MobileElementFinder (v1.0.3) [69] considering only ARGs located within the maximum transposon distance as defined for each organism in the database. The potential plasmid origin of contigs was evaluated with PlasFlow (v1.1) [70] and prophage-encoded ARGs were identified using VirSorter (v2.2.2) [71].

## 3. Results

**Figure 1:**
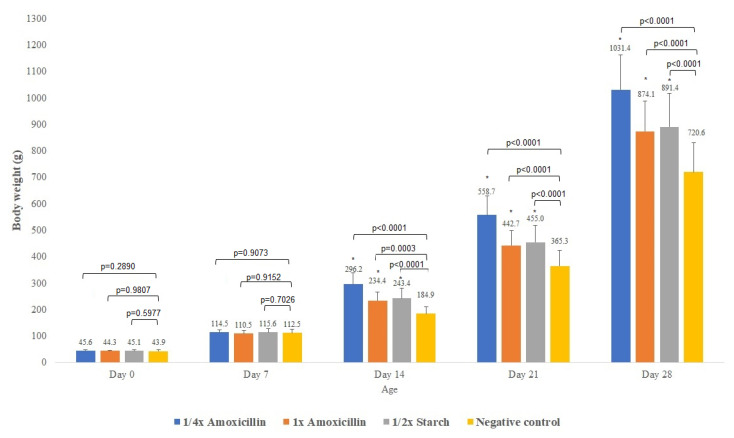
Average body weight of broiler chickens (mean ± SD) in each treatment group on days 0, 7, 14, 21, and 28 of the experiment. Exact *p*-values are displayed above the bars for each comparison with the untreated negative control group, based on one-way ANOVA. Asterisks (*) indicate statistically significant differences (*p* < 0.05) compared to the untreated negative control group.

Body weight gain was monitored through individual weekly weighing of all animals (Figure 1). By day 14 of the measurement schedule, significant differences in average body weight were observed among the treatment groups, indicating a measurable impact of the interventions on growth performance.

We performed a one-way ANOVA test to assess whether there were statistically significant differences in body weights between the treatment groups and the negative control group. Starting from day 14, all treatment conditions resulted in significantly higher body weights compared to the negative control group, indicating a positive effect of the interventions on growth performance.

Feed intake and feed conversion ratio were not monitored as these were outside the primary scope of the study; no mortality occurred in any of the experimental groups throughout the trial.

**Figure 2:**
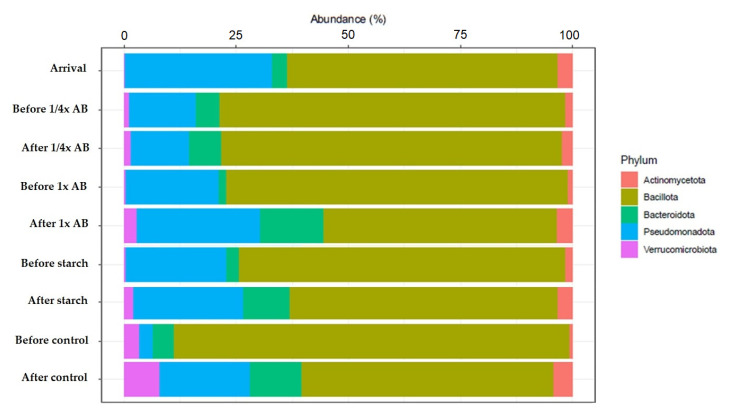
Shifts in gut microbiota composition at the phylum level across treatment groups, including day-old chicks, pre-treatment, and post-treatment samples. Only taxa with ≥1% relative abundance and ≥10% prevalence are shown. AB—antibiotic.

The quality parameters of samples prepared for sequencing are in the Supplementary Table S3. The exact results are given in the additional excel table. Following the sequencing of the fecal samples, we analyzed changes in gut microbiota composition across the different treatment groups. At the phylum level (Figure 2), Bacillota was the dominant phylum in all cases. In day-old chicks, Pseudomonadota represented the second most abundant phylum, although its relative abundance decreased substantially with age. Notably, with the exception of the group treated with the ¼× amoxicillin dose, the overall microbial composition shifted markedly across treatment groups—primarily due to a pronounced increase in the relative abundance of the Bacteroidota phylum.

**Figure 3:**
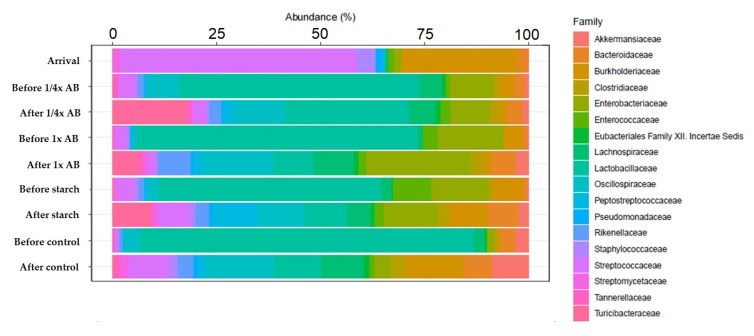
Changes in gut microbiota composition at the family level across treatment groups, including day-old chicks, pre-treatment, and post-treatment samples. Only taxa with ≥1% relative abundance and ≥10% prevalence are shown. AB—antibiotic.

We also examined gut microbiota composition at the family level (Figure 3). In day-old chicks, the dominant family was Streptococcaceae, which was gradually replaced by Lactobacillaceae as the birds aged. In response to the ¼× amoxicillin dose, Turicibacteraceae became the dominant family, while treatment with the full (1×) dose resulted in a marked increase in the relative abundance of Enterobacteriaceae.

**Figure 4:**
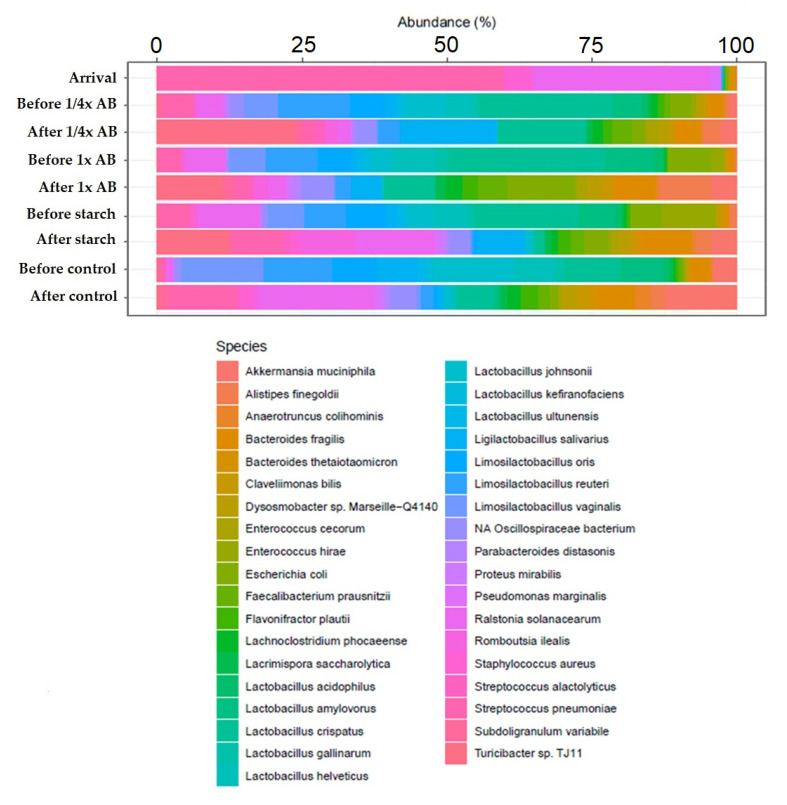
Species-level changes in gut microbiota composition across treatment groups, including day-old chicks, pre-treatment, and post-treatment samples. Only taxa with ≥1% relative abundance and ≥10% prevalence are shown. AB—antibiotic.

We further compared gut microbiota composition at the species level (Figure 4). In day-old chicks, the *Streptococcus* species were predominant. As the birds matured, the *Lactobacillus* species became the dominant taxa. However, in response to antibiotic treatment, we observed an increase in the relative abundance of the *Turicibacter* species, the *Subdoligranulum* variabile, and the *Streptococcus* species. In addition, the abundance of *Akkermansia muciniphila* and *Alistipes finegoldii* also increased substantially following treatment.

**Figure 5:**
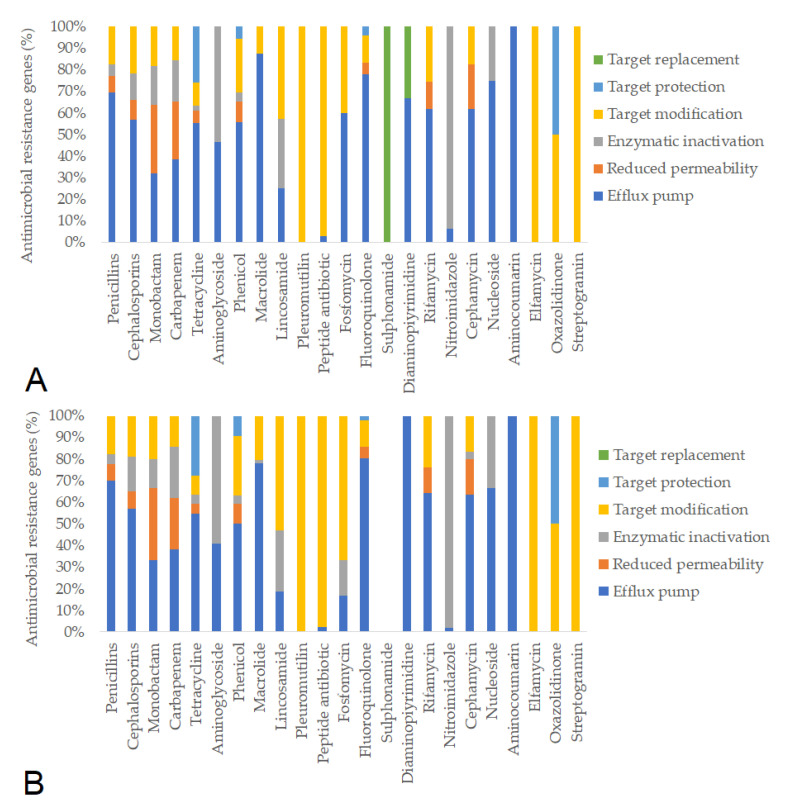
Antimicrobial resistance gene (ARG) profiles in the negative control groups, categorized by antimicrobial class and resistance mechanism, before (**A**) and after (**B**) treatment.

**Figure 6:**
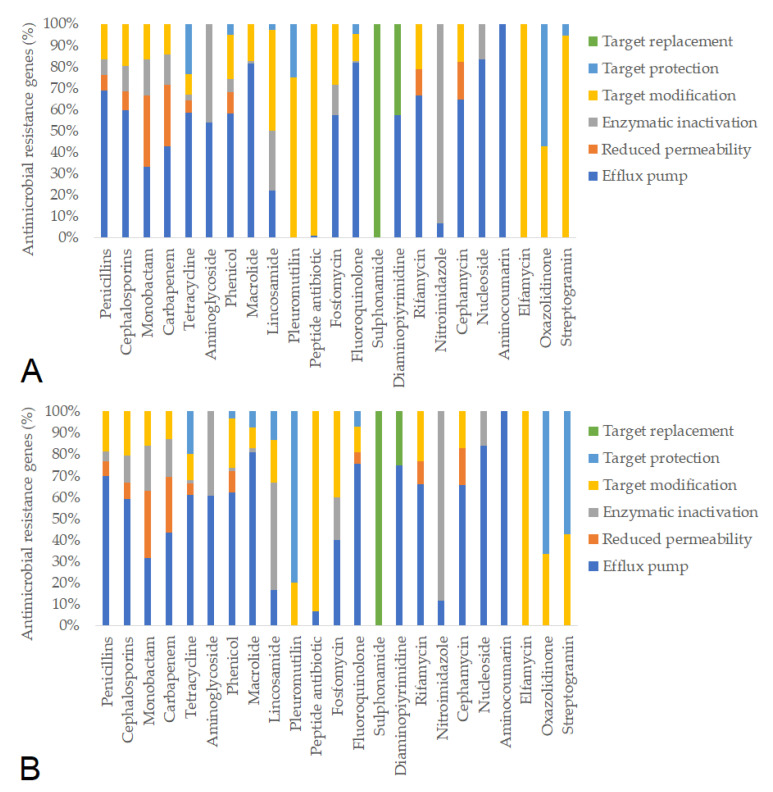
Antimicrobial resistance gene (ARG) profiles in the groups treated with the ¼× amoxicillin dose, categorized by antimicrobial class and resistance mechanism, before (**A**) and after (**B**) treatment.

**Figure 7:**
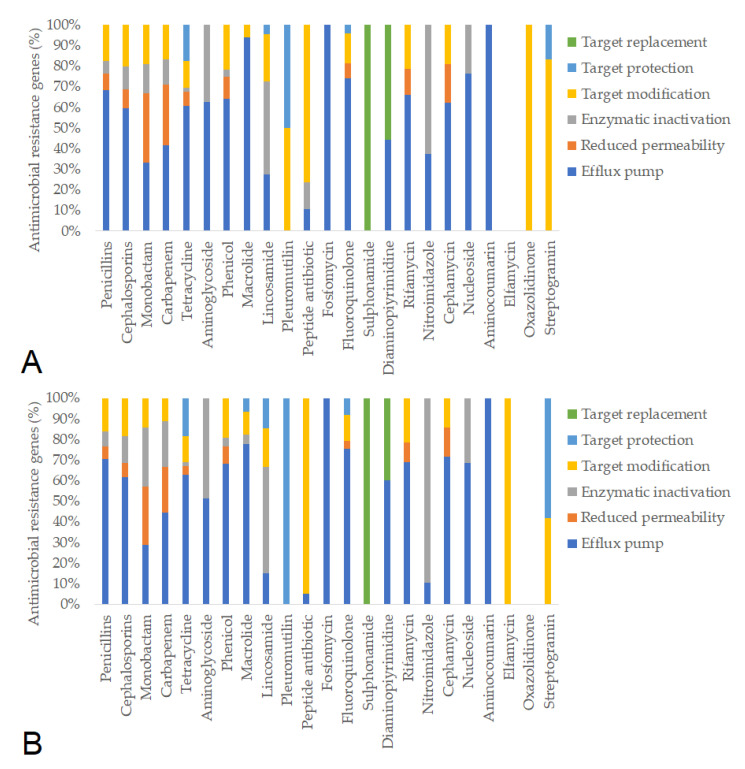
Antimicrobial resistance gene (ARG) profiles in the groups treated with the 1× amoxicillin dose, categorized by antimicrobial class and resistance mechanism, before (**A**) and after (**B**) treatment.

**Figure 8:**
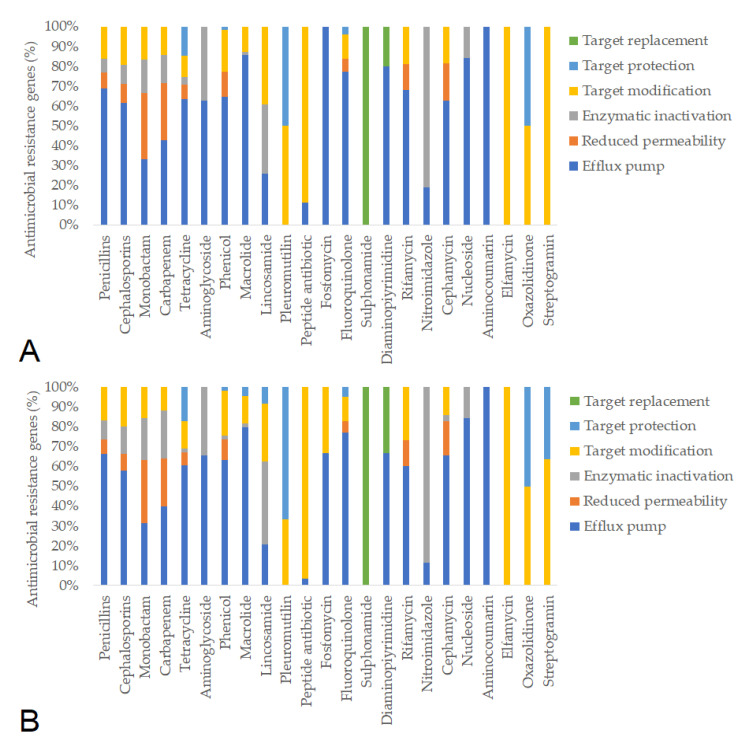
Antimicrobial resistance gene (ARG) profiles in the groups treated with the ½× starch dose, categorized by antimicrobial class and resistance mechanism, before (**A**) and after (**B**) treatment.

Subsequently, we identified the antimicrobial resistance gene (ARG) profiles from fecal metagenomes, considering only hits with >90% sequence coverage and identity. Based on these criteria, a total of 781 ARGs were detected in pre-treatment samples and 637 ARGs post-treatment. The resistance mechanisms associated with individual antimicrobial agents remained similar before and after feeding in the negative control group (Figure 5). In contrast, feeding the ¼× amoxicillin dose led to a marked reduction in the number of detected resistance genes (from 1386 to 1012), likely reflecting changes in gut microbiota composition (Figure 6). The full (1×) amoxicillin dose had minimal effect on the ARG profile (Figure 7), while the ½× starch vehicle caused a modest decrease (from 987 to 896 genes; Figure 8).

The presence of an ESBL-encoding gene (*CTX-M-1*) was detected exclusively in samples following treatment with the ¼× amoxicillin dose. The *TEM-1* and *TEM-135* genes, also associated with ESBL production, were detected at similar levels across all groups both before and after treatment.

Multiple genes associated with vancomycin resistance were also identified. Their abundance significantly decreased in response to the ¼× amoxicillin treatment, whereas an increasing trend was observed in all other treatment groups.

**Figure 9:**
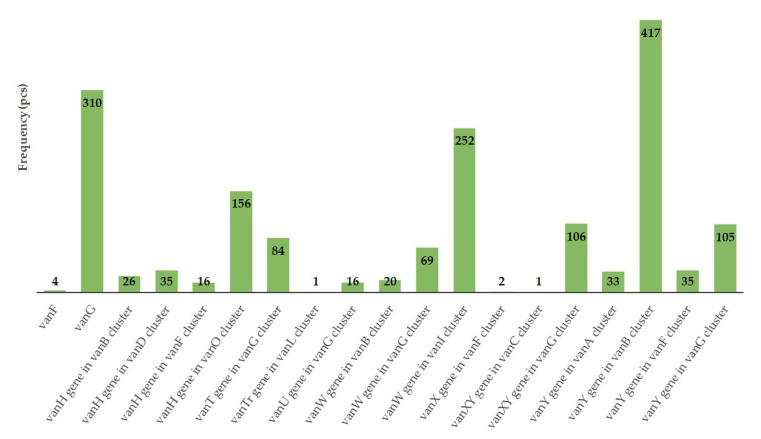
Frequency of total vancomycin resistance-associated antimicrobial resistance genes (ARGs) identified during the study.

We further analyzed the presence of specific gene clusters associated with vancomycin resistance. These included *vanY* (vanB cluster), *vanG* and *vanW* (vanI cluster), *vanH* (vanO cluster), *vanXY* (vanG cluster), and *vanY* (also mapped to the vanG cluster). Each of these gene variants was identified in over 100 instances (Figure 9). Vancomycin resistance genes were detected in all treatment groups both before and after treatment. The total number of *van* genes decreased in the ¼× amoxicillin group (334 to 170) but increased in the 1× amoxicillin (93 to 175), ½× excipient (97 to 151), and negative control (300 to 375) groups.

We also analyzed how the abundance of plasmid- and phage-associated resistance genes as well as the relative proportion of mobile genetic elements (MGEs) changed before and after treatment in each group (Table 2). A notable reduction in the number of plasmid-borne ARGs was observed in the groups treated with the ¼× amoxicillin dose. This group also exhibited the most pronounced decline in the relative abundance of MGEs. In contrast, the groups treated with the ½× starch vehicle showed a decrease in phage-associated ARGs, while the negative control groups displayed an overall increase in phage-borne resistance genes following the study period.

The abundance of ARGs associated with MGEs, plasmids, and phages was evaluated before and after treatment using the Wilcoxon matched-pairs signed-rank test (*n* = 3 per treatment group). Analyses were conducted separately for each treatment. No statistically significant differences were observed in ARG abundance between before-treatment and after-treatment samples in any case (*p* > 0.05 for all comparisons), exact *p*-values are provided in Supplementary Table S4.

## 4. Discussion

In this study, we evaluated the effects of a veterinary amoxicillin-containing formulation—authorized for use in poultry—administered to broiler chickens over a 10-day treatment period as recommended by the manufacturer. The study focused on comparing the impact of a reduced (¼×) and full (1×) dose of amoxicillin as well as the formulation’s excipient (½× starch) on weight gain, gut microbiota composition, and associated antimicrobial resistance gene (ARG) profiles. Compared to the negative control groups, we observed significant differences in body weight gain (*p* < 0.0001) in both treatment groups (¼× and 1× amoxicillin, and starch) on days 14, 21, and 28. These results are consistent with those of Singh et al. [54], who also reported significant improvements in body weight following penicillin administration in broilers relative to untreated controls.

At the phylum level, the gut microbiota in newly arrived day-old chicks was initially dominated by Bacillota (formerly Firmicutes, ~70%), followed by Pseudomonadota (formerly Proteobacteria, ~21%). Following administration of the ¼× amoxicillin dose, the relative abundance of Bacillota increased to 85%, while Pseudomonadota decreased to 9.25%. In contrast, the 1× dose resulted in a decline in Bacillota (to 59.25%) and a slight reduction in Pseudomonadota (15%). No substantial compositional shifts were observed following the administration of the ½× starch vehicle, and the microbiota of the negative control groups remained largely unchanged throughout the study. Similar dominance patterns of Firmicutes and Proteobacteria were reported by Ma et al. [72], who also described dynamic shifts in microbial diversity using Shannon index analyses.

At the family level, Lactobacillaceae represented 30–40% of the community prior to treatment, but declined to below 20% with increasing age and exposure to treatments. These shifts are in line with the findings of Wise and Siragusa [53], who described comparable age-dependent reductions in Lactobacillaceae abundance in broiler intestinal microbiota. At the species-level, the *Streptococcus* species predominated in day-old chicks, whereas the *Lactobacillus* species became more dominant as the birds grew. The observed decline in *Lactobacillus* spp. following amoxicillin treatment may be attributed to the broad-spectrum activity of beta-lactam antibiotics, which, although primarily targeting pathogenic bacteria, can also inadvertently suppress beneficial commensal populations. This disruption of gut microbiota equilibrium may impair colonization resistance and overall gut health, as similarly reported by Wise and Siragusa [53].

Overall, the ¼× amoxicillin treatment induced substantial shifts in gut microbiota composition, which coincided with a significant narrowing of the ARG repertoire. Notably, this effect was not observed with the 1× dose. The pronounced microbiota shifts and ARG narrowing observed under subtherapeutic (¼×) amoxicillin exposure may result from selective suppression of susceptible commensals without fully eliminating resistant strains, thereby reducing microbial diversity and ARG heterogeneity. Such low-dose antibiotic exposure has been shown to select for resistant bacteria even at concentrations far below therapeutic levels, by creating a niche where resistant subpopulations can thrive while susceptible microbes are suppressed [73]. In contrast, the 1× therapeutic dose may exert broader bactericidal effects that preserve a more diverse ARG pool through balanced microbial turnover and transient expansion of resistant subpopulations. This hypothesis aligns with evidence suggesting that sublethal antibiotic concentrations can perturb microbial communities in subtle yet resistance-favoring ways, including horizontal gene transfer and metabolic stress responses [74]. Minor reductions in ARG abundance were also observed in both the starch-treated and negative control groups, although these changes were not statistically significant. The presence of a substantial number of ARGs even in untreated animals—both before and after the experimental period—highlights the baseline prevalence of antimicrobial resistance that exists independently of antibiotic pressure [75], a conclusion echoed by Zhou et al. [76]. Moreover, agricultural studies have demonstrated that low-dose antimicrobial regimens, similar to the one applied here, can serve as powerful drivers of resistome expansion, especially in intensive animal production environments [77].

The observed reduction in ARG abundance following the administration of a subtherapeutic (¼×) dose of amoxicillin may be explained by several non-mutually exclusive ecological mechanisms. One plausible hypothesis is that low antibiotic concentrations exert weaker selective pressure, mildly inhibiting susceptible commensals without eradicating them, thereby preserving microbial diversity and limiting ecological niches for resistant strains. This retained competition may constrain both ARG proliferation and horizontal gene transfer. In contrast, the full therapeutic dose may induce broader microbial disruption, allowing resistant subpopulations and mobile elements to transiently expand and maintain ARG diversity. Prior studies have shown that sub-inhibitory antibiotic levels can influence quorum sensing, biofilm dynamics, and plasmid mobility, factors directly affecting ARG dissemination [73,74]. This paradoxical outcome aligns with earlier observations of selective bottlenecks under low-dose antibiotic exposure [73,74].

In our study, the *TEM-1* and *TEM-135* genes were traceable to *E. coli* and were detected in samples from all treatment groups. Hassen et al. reported the presence of the *TEM-1* gene in 25.8% of fecal and meat samples from chickens in a comprehensive investigation [78]. Furthermore, a study focusing on extended-spectrum β-lactamases (ESBLs) identified *TEM-1* as the most frequently occurring enzyme among Gram-negative bacteria in this category [79].

The *ampC* gene, associated with β-lactamase overproduction, was detected in 75% of all groups, and in all cases, the gene was identified as originating from *E. coli*. According to Tofani et al. [43], the ceca of broiler chickens can harbor up to 10^4^ CFU/g of *E. coli*, which frequently carry either ESBL genes or *ampC*.

Of particular concern was the widespread presence of 20 different vancomycin resistance genes, which were detected at high prevalence across numerous bacterial taxa within the gut microbiome. Strikingly, the relative abundance of these genes remained unchanged by any of the treatments. The most frequently detected vancomycin resistance genes in our study were *vanY* (*vanB* cluster), *vanG* and *vanW* (vanI cluster), *vanH* (*vanO* cluster), and *vanXY* and *vanY* (both *vanG* cluster). Each of these was identified in over 100 instances. In contrast, Gousai et al. [80] reported that in their survey of *Enterococcus faecium* isolates, the *vanA* gene was present in over 30% of samples, whereas *vanC*, *vanD*, *vanE*, and *vanG* were not detected. Despite antibiotic administration, vancomycin resistance genes remained detectable in all treatment groups. The total number of *van* genes decreased in the ¼× amoxicillin group (from 334 to 170) but increased in the 1× group (93 to 175), the ½× excipient group (97 to 151), and the negative control (300 to 375). This suggests a persistent reservoir of vancomycin resistance genes within the gut microbiome, independent of treatment type.

Among the β-lactam resistance mechanisms, efflux pump genes were the most prevalent across all groups, both before and after treatment. Target modification was the second most common mechanism overall. The third most frequent mechanism varied by group: in the ¼× amoxicillin and negative control groups, reduced membrane permeability ranked third in both pre- and post-treatment samples. However, in birds treated with 1× amoxicillin or starch, reduced permeability was the third most common mechanism before treatment, while antibiotic inactivation took its place after the treatment period. These findings align partially with the results of Fernández et al. [81], who reported antibiotic inactivation and target modification as the primary β-lactam resistance mechanisms in poultry microbiota.

Taken together, our findings show the selective pressure exerted by antibiotics on gut microbiota, which in turn strongly influences the structure and abundance of the associated antimicrobial resistome. Future investigations should explore the effects of additional antibiotic classes and employ larger sample sizes combined with high-throughput sequencing technologies to provide broader ecological insight into the consequences of antibiotic use in livestock production systems. Based on our findings, we hypothesize that subtherapeutic antibiotic dosing may preferentially suppress sensitive commensal taxa such as *Lactobacillus* spp., thereby reducing microbial competition and allowing the expansion of more resistant or opportunistic strains. Furthermore, the observed narrowing of the ARG repertoire under low-dose amoxicillin treatment may reflect a selective bottleneck effect, whereby only a limited subset of resistance genes provides a competitive advantage in this altered gut environment. This could be driven by changes in host immune signaling, horizontal gene transfer rates, or shifts in metabolite availability induced by antibiotic pressure. These hypotheses merit targeted exploration using metagenomic, transcriptomic, and metabolomic approaches in future studies.

## 5. Conclusions

Our findings confirm that even short-term administration of an amoxicillin-containing formulation at a clinical dose has a significant impact on the gut microbiota of poultry, particularly in terms of ARG abundance. The full dose did not significantly change the total amount of ARG, but it may promote the emergence of specific ARGs (such as ESBL genes). The association of ARGs with MGEs poses an additional risk, as it may facilitate horizontal gene transfer between bacterial species, including potential pathogens. These pathogens could easily be transmitted between livestock and potentially zoonotically, entering the human food chain.

The results of this study highlight the need for deeper systematic research into how antibiotics affect microbiota and resistome. They also emphasize the importance of promoting targeted and responsible antibiotic use in intensive livestock production systems. Moving forward, regular and regional monitoring of gut resistomes will be essential to inform effective strategies for mitigating antimicrobial resistance, in line with the principles of the One Health approach.

## Figures and Tables

**Table 1:** Daily administration scheme of the amoxicillin-containing veterinary formulation across treatment groups over the 10-day period. The table includes both the full (1×) and quarter (¼×) therapeutic doses, as well as the placebo used in the control groups.

Day of Treatment	Average BW (kg)	Group Size (*n*)	Dose 1× (g)	Dose ¼× (g)	Placebo Starch ½× (g)
1	0.4	30	0.48	0.12	0.24
2	0.6	30	0.72	0.18	0.36
3	0.6	30	0.72	0.18	0.36
4	0.6	30	0.72	0.18	0.36
5	0.8	30	0.96	0.24	0.48
6	0.8	30	0.96	0.24	0.48
7	0.9	30	1.08	0.27	0.54
8	0.9	30	1.08	0.27	0.54
9	1	30	1.2	0.3	0.6
10	1	30	1.2	0.3	0.6

BW—body weight.

**Table 2:** Prevalence of plasmid- and phage-associated antimicrobial resistance genes, and mobile genetic elements (MGEs), identified before and after treatment across all experimental groups (mean ± standard deviation).

Treatment	¼× Amoxicillin		1× Amoxicillin		½× Starch		Negative Control	
	Before	After	Before	After	Before	After	Before	After
Plasmid	105 (±8.9)	66 (±3.2)	66 (±6.4)	64 (±7.8)	59 (±0.6)	58 (±5.0)	110 (±10.0)	89 (±6.0)
Phage	13 (±1.7)	13 (±2.0)	5 (±3.1)	6 (±3.1)	12 (±5.6)	7 (±6.7)	5 (±3.8)	17 (±8.6)
MGE *	34 (±1.5)	19 (±3.1)	33 (±2.0)	36 (±6.6)	31 (±4.2)	22 (±3.8)	24 (±9.5)	27 (±7.6)

* MGE—mobile genetic element.

## Data Availability

The datasets used and/or analyzed during the current study are available from the corresponding author on reasonable request. The sequencing files are available at the https://www.ncbi.nlm.nih.gov/bioproject/PRJNA1268717, accessed on 28 May 2025.

## Supplementary Materials

The following supporting information can be downloaded at: https://www.mdpi.com/article/10.3390/ani15131944/s1. Table S1: Environmental parameters required during the rearing period of broiler chickens. Table S2: The main ingredients of broiler feed. Table S3: Quality parameters of samples prepared for sequencing. Table S4: Statistical comparison of the prevalence of plasmid-associated, phage-associated, and mobile genetic element (MGE)-linked antimicrobial resistance genes before and after treatment in each experimental group. The exact results are given in the additional excel table.

## Abbreviations

The following abbreviations are used in this manuscript:

AMR	Antimicrobial resistance
ARG	Antimicrobial resistance gene
ESBL	Extended-spectrum beta-lactamase
MDR	Multidrug-resistant
MGE	Mobile genetic element
