# Supplementary material for: Amoxicillin Resistance: An In Vivo Study on the Effects of an Approved Formulation on Antibiotic Resistance in Broiler Chickens
Source: Animals (Basel). 2025 Jul 1;15(13):1944. doi: 10.3390/ani15131944 (PMC12249013; doi:10.3390/ani15131944)
Supplement: Supplementary file 1 [file animals-15-01944-s001.zip › animals-3726996-supplementary.pdf]

Supplementary Table S1. *Environmental parameters required during the rearing period of broiler chickens*

Day	Temperature °C	Humidity %	Length of lighting (hours)
1	30.8	60-70%	23
2	30.8	60-70%	23
3	28.9	60-70%	23
4	28.8	60-70%	23
5	28.9	60-70%	23
6	27.7	60-70%	23
7	27.7	60-70%	22
8	27.7	60-70%	21
9	26.7	60-70%	20
10	26.7	60-70%	19
11	26.7	60-70%	18
12	25.7	60-70%	18
13	25.7	60-70%	18
14	25.7	60-70%	18
15	24.8	60-70%	18
16	24.8	60-70%	18
17	24.8	60-70%	18
18	23.6	60-70%	18
19	23.6	60-70%	18
20	23.6	60-70%	18
21	22.7	60-70%	18
22	22.7	60-70%	18
23	22.7	60-70%	18
24	21.7	60-70%	18
25	21.7	60-70%	18
26	21.7	60-70%	18
27	20.7	60-70%	18
28	20.7	60-70%	18

Supplementary Table S2. *The main ingredients of broiler feed*

**Component**	**Starter**	**Grower**
	**0-14 Days**	**15-28 Days**
Wheat	40.0 %	40.0 %
Soy	29.2 %	31.2 %
Corn	24.8 %	23.8 %
Sunflower seed oil	2.0 %	1.0 %
Premix[1]	4.0 %	4.0 %
AMEn[2] poultry	12.03 MJ/kg	13.00 MJ/kg
Dry material	89.54%	88.53%
Crude protein	20.96%	20.49%
Crude fat	6.00%	6.32%
Crude fiber	2.86%	3.17%
Crude ash	6.07%	5.84%
Lysine	1.25%	1.20%
Methionine	0.54%	0.55%
Calcium	1.05%	0.85%
Phosphor	0.75%	0.66%
Sodium	0.16%	0.17%

[1] Szőlőfürt Szövetkezet 4% Premix

[2] apparent metabolizable energy corrected to zero nitrogen retention

Supplementary Table S3 *Quality parameters of samples prepared for sequencing*

No	Index 1	Index 2	Samples		Concentration (µg/mL)	to 0.2 (multiples) (µg/mL)	2 µL+ x H_2O (µg/mL)	Library concentration (µg/mL)
1	N726 - CCTAAGAC	S515 - TTCTAGCT	61-63	arriving	0.904	4.52	7.04	3.82
2	N727 - CGATCAGT	S515 - TTCTAGCT	114-116	arriving	1.42	7.1	12.2	1.78
3	N728 - TGCAGCTA	S515 - TTCTAGCT	1-3	before treatment	0.218	1.09	0.18	1.1
4	N729 - TCGACGTC	S515 - TTCTAGCT	11-13	before treatment	0.354	1.77	1.54	2.7
5	N716 - ACTCGCTA	S516 - CCTAGAGT	14-16	before treatment	1.34	6.7	11.4	1.48
6	N718 - GGAGCTAC	S516 - CCTAGAGT	24-26	before treatment	0.212	1.06	0.12	2.08
7	N719 - GCGTAGTA	S516 - CCTAGAGT	34-36	before treatment	0.268	1.34	0.68	6.04
8	N720 - CGGAGCCT	S516 - CCTAGAGT	41-43	before treatment	0.568	2.84	3.68	4.4
9	N721 - TACGCTGC	S516 - CCTAGAGT	47-50	before treatment	0.594	2.97	3.94	1.09
10	N722 - ATGCGCAG	S516 - CCTAGAGT	51-53	before treatment	0.646	3.23	4.46	1.11
11	N723 - TAGCGCTC	S516 - CCTAGAGT	67-70	before treatment	0.486	2.43	2.86	1.94
12	N724 - ACTGAGCG	S516 - CCTAGAGT	74-76	before treatment	0.46	2.3	2.6	2.16
13	N726 - CCTAAGAC	S516 - CCTAGAGT	81-83	before treatment	0.56	2.8	3.6	1.67
14	N727 - CGATCAGT	S516 - CCTAGAGT	84-86	before treatment	1.49	7.45	12.9	1.04
15	N728 - TGCAGCTA	S516 - CCTAGAGT	92-94	before treatment	0.784	3.92	5.84	6.28
16	N729 - TCGACGTC	S516 - CCTAGAGT	97-100	before treatment	1.98	9.9	17.8	5.72
17	N716 - ACTCGCTA	S517 - GCGTAAGA	104-06	before treatment	0.23	1.15	0.3	1.71
18	N718 - GGAGCTAC	S517 - GCGTAAGA	107-110	before treatment	0.338	1.69	1.38	0.896
19	N719 - GCGTAGTA	S517 - GCGTAAGA	1-3	after treatment	5.78	28.9	55.8	2.02
20	N720 - CGGAGCCT	S517 - GCGTAAGA	11-13	after treatment	5.14	25.7	49.4	3.36
21	N721 - TACGCTGC	S517 - GCGTAAGA	14-16	after treatment	1.01	5.05	8.1	5.44
22	N722 - ATGCGCAG	S517 - GCGTAAGA	24-26	after treatment	5.64	28.2	54.4	5.56
23	N723 - TAGCGCTC	S517 - GCGTAAGA	37-40	after treatment	1.15	5.75	9.5	2.57
24	N724 - ACTGAGCG	S517 - GCGTAAGA	47-50	after treatment	5.44	27.2	52.4	1.79
25	N726 - CCTAAGAC	S517 - GCGTAAGA	51-53	after treatment	2.64	13.2	24.4	2.06

26	N727 - CGATCAGT	S517 - GCGTAAGA	54-56	after treatment	2.44	12.2	22.4	3.68
27	N728 - TGCAGCTA	S517 - GCGTAAGA	64-66	after treatment	0.218	1.09	0.18	1.26
28	N729 - TCGACGTC	S517 - GCGTAAGA	71-73	after treatment	1.57	7.85	13.7	2.92
29	N716 - ACTCGCTA	S518 - CTATTAAG	77-80	after treatment	1.27	6.35	10.7	1.69
30	N718 - GGAGCTAC	S518 - CTATTAAG	84-86	after treatment	0.632	3.16	4.32	3.91
31	N719 - GCGTAGTA	S518 - CTATTAAG	91-93	after treatment	8.86	44.3	86.6	3.42
32	N720 - CGGAGCCT	S518 - CTATTAAG	97-100	after treatment	1.7	8.5	15	2.08
33	N721 - TACGCTGC	S518 - CTATTAAG	101-103	after treatment	1.22	6.1	10.2	2.66
34	N722 - ATGCGCAG	S518 - CTATTAAG	114-116	after treatment	1.2	6	10	3.28

Supplementary Table S4 *Statistical comparison of the prevalence of plasmid-associated, phage-associated, and mobile genetic element (MGE)-linked antimicrobial resistance genes before and after treatment in each experimental group. P-values were calculated using the Wilcoxon signed-rank test (n = 3 per group).*

**Treatment**	**¼× amoxicillin**		**1× amoxicillin**		**½ × starch**		**Negative control**	
	**Before**	**After**	**Before**	**After**	**Before**	**After**	**Before**	**After**
	p-values							
Plasmid	0.1000		0.1212		1.000		0.1000	
Phage	1.0000		0.7000		0.4000		0.2000	
MGE*	0.1000		0.5066		0.1000		1.000	

*Mobile Genetic Elements
